# Low-dose ketamine infusion to facilitate opioid tapering in chronic non-cancer pain with opioid-use disorder: a historical cohort study

**DOI:** 10.1136/rapm-2023-105035

**Published:** 2024-03-18

**Authors:** Antoine Elyn, Anne Roussin, Cécile Lestrade, Nicolas Franchitto, Bénédicte Jullian, Nathalie Cantagrel

**Affiliations:** 1 Chronic Pain Center, University Hospital of Toulouse, Toulouse, France; 2 General and Family Medicine University Department, University of Toulouse III - Paul Sabatier, Toulouse, France; 3 RECaP F-CRIN - Réseau national de Recherche en Épidémiologie Clinique et en Santé Publique, Inserm, Toulouse, France; 4 Clinical Pharmacology, University Hospital of Toulouse, Toulouse, France; 5 INSERM UMR1295, Pharmaco-épidémiologie, University of Toulouse III - Paul Sabatier, Toulouse, France; 6 University of Medicine, University of Toulouse III - Paul Sabatier, Toulouse, France; 7 Clinical Addictology Center, University Hospital of Toulouse, Toulouse, France; 8 INSERM UMR1295, EQUITY “Embodiment, social inequalities, lifecourse epidemiology, cancer and chronic diseases, interventions, methodology”, University of Toulouse III - Paul Sabatier, Toulouse, France

**Keywords:** CHRONIC PAIN, Opioid-Related Disorders, Analgesics, Opioid

## Abstract

**Background:**

Long-term opioid use is associated with pharmacological tolerance, a risk of misuse and hyperalgesia in patients with chronic pain (CP). Tapering is challenging in this context, particularly with comorbid opioid-use disorder (OUD). The antihyperalgesic effect of ketamine, through N-methyl-D-aspartate (NMDA) antagonism, could be useful. We aimed to describe the changes in the dose of opioids consumed over 1 year after a 5-day hospitalisation with ketamine infusion for CP patients with OUD.

**Methods:**

We performed a historical cohort study using a medical chart from 1 January 2014 to 31 December 2019. Patients were long-term opioid users with OUD and CP, followed by the Pain Center of the University Hospital of Toulouse, for which outpatient progressive tapering failed. Ketamine was administered at a low dose to initiate tapering during a 5-day hospitalisation.

**Results:**

59 patients were included, with 64% of them female and a mean age of 48±10 years old. The most frequent CP aetiologies were back pain (53%) and fibromyalgia (17%). The baseline opioid daily dose was 207 mg (±128) morphine milligram equivalent (MME). It was lowered to 92±72 mg MME at discharge (p<0.001), 99±77 mg at 3 months (p<0.001) and 103±106 mg at 12 months. More than 50% tapering was achieved immediately for 40 patients (68%), with immediate cessation for seven patients (12%). 17 patients were lost to follow-up.

**Conclusions:**

A 5-day hospitalisation with a low-dose ketamine infusion appeared useful to facilitate opioid tapering in long-term opioid users with CP and OUD. Ketamine was well tolerated, and patients did not present significant withdrawal symptoms. Prospective and comparative studies are needed to confirm our findings.

**WHAT IS ALREADY KNOWN ON THIS TOPIC**

**WHAT THIS STUDY ADDS**

- A 5-day in-hospital low-dose infusion of ketamine allowed more than 50% of the opioid dose decrease for 40 patients (68%) at discharge and was maintained over time without significant opioid withdrawal symptoms or ketamine adverse effects.

**HOW THIS STUDY MIGHT AFFECT RESEARCH, PRACTICE OR POLICY**

- Low-dose ketamine infusions could help initiate opioid tapering in the context of chronic non-cancer pain with opioid-use disorder.

## Introduction

 Long-term use of opioids is common among people with chronic pain (CP) and has more than doubled in recent years.^1^ Yet, their effectiveness in relieving CP remains controversial, with significant adverse effects and pharmacological tolerance to the analgesic effects. This can lead to dose escalation and opioid-induced hyperalgesia, but also to the risk of addiction and high morbi-mortality.^2 3^ National guidelines recommend tapering opioid analgesics in this population.^4 5^ A reduction in hyperalgesia is proven, with a dose-dependent effect.^6^ Progressive tapering with patient education can be a successful strategy without increasing pain.^7 8^ However, this is a challenge for both patients and practitioners, especially in the context of comorbid opioid-use disorder (OUD).

Due to a wide range of pharmacological actions, ketamine appears as an interesting option to facilitate opioid tapering in the context of CP with OUD. It interacts with various neurological pathways (serotonin, glutamate, opioid, adrenergic, dopaminergic, etc.). Ketamine analgesic effects are well documented, particularly for neuropathic pain, and they permit reducing the opioid requirement.^9 10^ In the context of postoperative pain, Nielsen and colleagues (2017 and 2019) demonstrated the potential benefit of intraoperative low-dose ketamine use during back surgery on immediate and long-term opioid dose consumption.^11 12^ The N-Methyl-D-Aspartate (NMDA)-antagonist activity of ketamine explains it potential to reduce opioid-induced hyperalgesia.^13^ This phenomenon is frequently reported among chronic opioid users.^2^ As a treatment for opioid withdrawal and as a treatment for OUD, only case reports and a small series suggest the potential value of ketamine to prevent withdrawal symptoms.^14 15^ However, there is only little evidence to support the use of ketamine for tapering opioids in patients with CP and OUD.^16^

The aim of our study was to describe the benefits of an initial infusion of low-dose ketamine during a 5-day hospitalisation on changes in opioid consumption and the adverse effects of ketamine. Our aim was also to investigate factors potentially associated with the initial opioid decrease and failure to sustain tapering over a 3-month follow-up.

## Methods

### Study population

We included patients hospitalised for a low-dose ketamine infusion to facilitate opioid tapering between 1 January 2014 and 31 December 2019. Patients were followed in the CP centre of Toulouse University Hospital. These patients suffered from CP with the long-term use of opioid analgesics and the failure of progressive opioid tapering. They were all fulfilling the Diagnostic and Statistical Manual of Mental Disorders, fifth edition (DSM-5 criteria of OUD. Failure of progressive opioid tapering was defined as an impossibility to decrease opioid consumption in the outpatient setting, given uncomfortable withdrawal symptoms. Patients with very high opioid requirements and intense craving symptoms were not included in our study when opioid substitution treatment (methadone) was chosen. Patients who presented counterindications for the use of ketamine were not included (eg, chronic liver disease or disturbed hepatic function (a blood test prior to hospitalisation was performed to verify transaminase levels), psychotic disorders, long QT syndrome or heart rhythm disorders).

### Ethical and reglementary obligations

Our study was assessed and approved in conformity with retrospective methodology (MR-004). This methodology is validated by the French National Data Commission (Commission Nationale de l’Informatique et des Libertés (CNIL)) and Toulouse University Hospital. There was no requirement for written, informed consent. The data were collected from the medical charts.

### Hospitalisation for opioid tapering with ketamine administration

Tapering was initiated during a 5-day hospitalisation in the pain department. Patients underwent psychological assessment, followed by psychoeducation on CP mechanisms and OUD. Patients were monitored daily by a multidisciplinary medical team (an anesthesiologist, a pain specialist and a psychiatrist). Ketamine (ATC code N01A×03) was administered intravenously with a titration followed by a 5-day continuous infusion. During the titration phase, slow boluses of 0.15 mg/kg were administered every 30 min to achieve pain relief (numerical rating pain scale less than or equal to 3 over 10).^17^ A maximum of three boluses could be administered (for a total dose of 0.45 mg/kg). After titration, the total dose of titrated ketamine was administered each day with an electric syringe in 24-hour periods during the entire hospitalisation, so the ketamine infusion rate was between 0.15 mg/kg/24 hours and 0.45 mg/kg/24 hours. Before the first administration of ketamine, an ECG was performed, and vital signs (pulse and blood pressure) were measured. During the titration phase, blood pressure was monitored every 30 min. A blood test was performed to check for potential hepatic contraindications prior to the first ketamine administration. Adverse effects were monitored during each phase. In the event of an adverse effect, administration was immediately suspended for 1 hour. In case of complete resolution, continuous infusion was resumed if the patient agreed. No other analgesics were used.

### Rapid-onset opioid tapering strategy

The opioid tapering strategy was a daily joint decision by the patient and the pain physician. The first step was to stop immediate-release opioids, in most instances, on the second day.^18^ Sustained-release opioids were then progressively decreased according to withdrawal symptoms as expressed by the patient. The Clinical Opiate Withdrawal Scale (COWS) was routinely assessed each day.^19^ In cases of significant withdrawal symptoms, opioids were stabilised. The tapering strategy was not predefined and was discussed daily.

### Outpatient follow-up

All patients were discharged on the fifth day. After discharge, patients were reassessed by the medical team at 1, 3, 6 and 12 months during dedicated consultations. The general practitioner was the only opioid prescriber after discharge. Dose adaptations were discussed and validated with the patient. When possible, complete opioid discontinuation was defined as a patient-specific goal.

### Outcomes

We described the changes in opioid dose over time in morphine milligram equivalent (MME). MMEs were calculated using validated equianalgesic ratios.^20^ The initial opioid tapering was consistently described as a percentage of the initial dose (%). It was defined as the daily opioid dose at discharge compared with the initial opioid dose. Failure to sustain opioid tapering over the 3 months was defined as a reduction of less than 30% of the initial opioid dose, considering our data distribution and specific guidelines (ie, 10% per month when opioids are taken for years).^21^

### Covariates

We considered socioeconomic and clinical variables: age, sex, weight, rurality, marital status, having children, employment status and severe OUD (≥6 symptoms among the DSM-5 criteria,^22^ non-opioid substance use disorders (tobacco, alcohol or cannabis), as well as other psychological comorbidities (eg, depression and anxiety). Both substance use disorders and psychological comorbidities were patient- reported. We also considered the pain phenotype: aetiology, the context of pain apparition (eg, postoperative), the mechanism (nociceptive, peripheral or central neuropathic or nociplastic) and widespread localisation. We collected data on opioid use: length of exposure (<5 years or ≥5 years, considering that very long-term exposure is known to decrease the success rate of opioid taper), high dosage (≥100 MME, given for increased risk of overdose),^23^ opioid types (tramadol or codeine (ATC code N02AJ06/J13 or N02A×02), morphine (ATC code N02AA01), oxycodone (ATC code N02AA05) and fentanyl (ATC code N02AB03)), route of administration and method of release (immediate or sustained). Other medications’ use was also considered: benzodiazepines (ATC code N05BA), gabapentinoids (ATC code N03A×12/16) and antidepressants (ATC code N06AA04/09 or N06A×16/17/18).

### Statistical analysis

To describe the benefit of our strategy, we first compared opioid consumption from baseline to each time-point: at discharge and 3 months after. A pairwise t-test was used, with a Bonferroni adjustment for multiple comparisons. The significance threshold was defined at p<0.05. Potential associations with initial opioid tapering amplitude were investigated by one-factor analysis of variance for categorical covariates and by non-parametric Spearman’s rank correlation coefficients for continuous covariates. Potential associations with failure to sustain opioid tapering at 3 months were studied using the χ^2^ test for categorical covariates (or Fisher’s exact test if conditions were not fulfilled) and the t-test for continuous covariates (or Wilcoxon-Mann-Whitney in cases of non-normal distribution or variance’s heterogeneity). All analyses were performed with Stata V.18.0 (StataCorp, College Station, TX, USA).

## Results

### Study population

**Figure 1:**
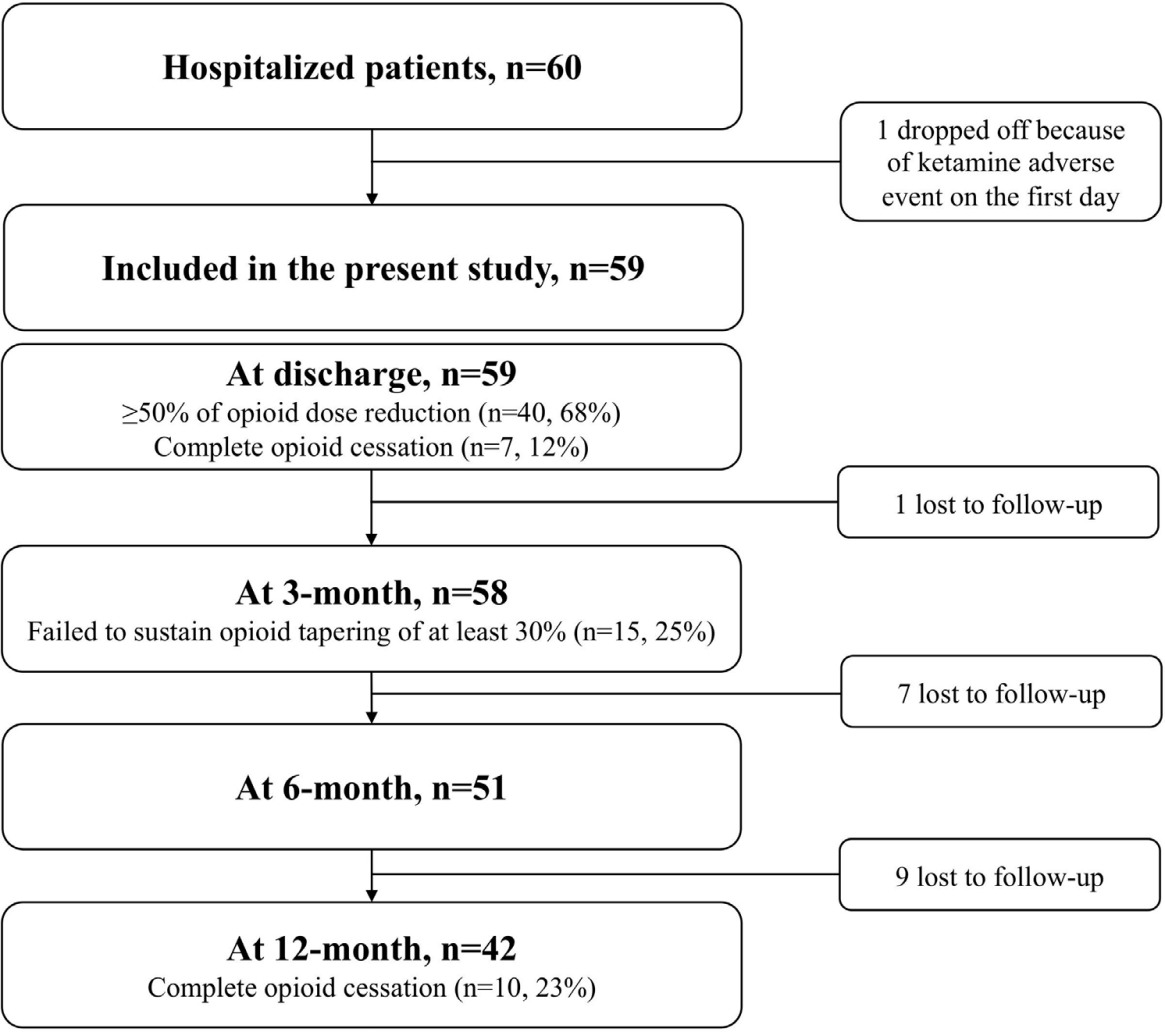
Flow diagram of the study.

Among 60 hospitalised patients, opioids were tapered between 1 January 2014 and 31 December 2019 in 59 hospitalised patients. One patient was excluded from the study due to the adverse effect of ketamine on the titration phase (figure 1). The mean age was 48±10 years (table 1). Sixty-four per cent (n=38) was female. The prevalence of psychiatric comorbidities was 83%. Almost half of the study population (48%) had a substance use disorder, such as alcohol (12%) or cannabis (10%). The main pain disorders were back pain (n=33) and fibromyalgia (n=10). Postoperative pain concerned 21 patients (31.6%). A neuropathic component was identified for 70%. Widespread pain was found in half of the sample, reflecting central sensitisation. Twenty patients had been using opioids for more than 5 years. Most patients were consuming high doses (≥100 MME, n=44, 75%). The main opioid types were oxycodone (71%) and morphine (15%). Fentanyl was used by 12%, and 7% used tramadol or codeine. One-quarter (n=14) of the population presented with a severe OUD. At the end of the follow-up, 17 patients were lost to follow-up, mainly women, and in the context of postoperative pain.

**Table 1:** Descriptive characteristics of the study population at baseline

	All patients
Sample size, n	59
Sociodemographic characteristics	
Mean age (SD) in years	48.42 (10.43)
Female sex, n (%)	38 (64.4)
Currently working, n (%)	9 (15.3)
Mean weight (SD) in kg	74.25 (19.63)
Non-opioid substance use disorders	
Tobacco, n (%)	25 (42.4)
Alcohol, n (%)	7 (11.9)
Cannabis, n (%)	6 (10.2)
History of mental disorder, n (%)	40 (67.8)
Pain characteristics	
Pain aetiology, n(%)	
Chronic back pain	31 (52.5)
Fibromyalgia	10 (17.0)
Other	18 (30.5)
Postoperative pain, n (%)	21 (35.6)
Peripheral neuropathic pain, n (%)	30 (50.8)
Widespread pain, n (%)	29 (49.2)
Opioid treatment	
>5 years of opioid exposure, n (%)	20 (33.9)
Severe opioid use disorder, n (%)	14 (23.7)
Use of tramadol or codeine only, n (%)	4 (6.8)
Use of oxycodone, n (%)	42 (71.2)
Use of transcutaneous fentanyl, n (%)	7 (11.9)
Use of oral route only, n (%)	43 (72.9)
Use of slow-release only, n (%)	10 (16.9)
Mean daily dose (SD) in mg/24 hours	206.75 (128.10)
High daily dose (≥100 morphine milligram equivalent, mg/24 hours), n (%)	44 (74.6)
Current use of benzodiazepine, n (%)	30 (50.8)
Current use of gabapentinoid, n (%)	29 (49.2)
Current use of antidepressant, n (%)	46 (78.0)

MME, Morphine Milligram Equivalent; n, number; OUD, Opioid-Use Disorder; SD, Standart Deviation.

### Opioid tapering and change over time

**Figure 2:**
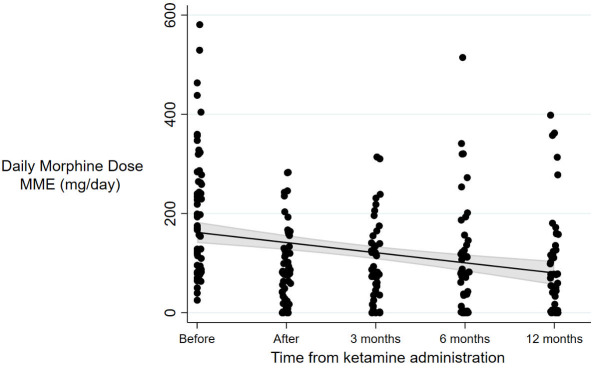
Opioid dose changes over time in mg per day of MME. The grey area represents the 95% CI; black line represents the evolution of the mean MME, and the black points represent the individual values at each time point. Opioid dose changes over time in mg per day of MME, before the ketamine administration, at discharge from the hospital (after) and then at 3, 6 and 12 months. The mean opioid dose between baseline and after ketamine was significantly lower (p<0.001). MME, morphine milligram equivalent.

Before hospitalisation, the mean daily opioid dose was established at 207 mg (±128) MME (figure 2). At discharge, the opioid dose was 92 mg (±72) MME and the mean reduction from baseline was established at 115.1 mg with a 95% CI (69.4–160.7) (p<0.001). The initial mean opioid dose reduction was calculated at 57±24%. An immediate dose reduction of ≥50% was obtained for 40 patients (68%) and seven patients (12%) were opioid-free at discharge. At discharge, none of the patients presented significant withdrawal symptoms and COWS remained ≤12/48 (with a minimum of 3, up to 9). At 3 months, the opioid dose was 99±77 mg MME (n=58) and the mean reduction from baseline was established at 97.5 mg with a 95% CI (51.7–143.3) (p<0.001). Fifteen patients (25%) failed to sustain tapering of at least 30% of the initial dose. Doses were estimated at 110±110 mg at 6 months (n=51) and at 103±106 mg at 12 months (n=42). Among the 42 patients with a complete 1-year follow-up, 10 patients (23%) discontinued opioids.

### Ketamine administration and adverse events

After initial titration, patients received a mean ketamine infusion rate of 0.31±0.15 mg/kg/day. Two patients reported adverse events that led to the discontinuation of ketamine. The first patient developed dizziness without hypotension, which resolved 1 hour later. Ketamine was then readministered at patient’s request without any problem. The second patient experienced nausea after the second bolus, and ketamine administration was discontinued. This patient dropped out of the study. None of the patients reported neuropsychological or psychiatric symptoms.

### Factors associated with the amplitude of initial opioid tapering

The use of tramadol or codeine was associated with better initial opioid tapering (table 2). Alcohol use disorder was also associated with better tapering. In contrast, psychological comorbidities and higher initial opioid daily doses were associated with poorer tapering.

**Table 2:** Bivariate analysis regarding initial opioid dose reduction, defined as a continuous variable with percentage decrease

	All patients	Initial opioid dose reduction in percentage	
	Mean (SD) or n (%)	Mean (SD) or corr. coef.	P value*†
Sample size, n	59		
Sociodemographic characteristics			
Mean age (SD) in years	48.42 (10.43)	−0.0237	0.86
Sex			
Female	38 (64.4)	0.56 (0.26)	0.44
Male	21 (35.6)	0.61 (0.20)	
Currently working			
Yes	9 (15.3)	0.57 (0.24)	0.98
No	50 (84.7)	0.58 (0.24)	
Non-opioid substance use disorders			
Tobacco			
Yes	25 (42.4)	0.62 (0.19)	0.26
No	34 (57.6)	0.54 (0.27)	
Alcohol			
Yes	7 (11.9)	0.76 (0.24)	*0.03*
No	52 (88.1)	0.55 (0.23)	
Cannabis			
Yes	6 (10.2)	0.50 (0.13)	0.43
No	53 (89.8)	0.58 (0.25)	
History of mental disorder			
Yes	40 (67.8)	0.53 (0.20)	*0.03*
No	19 (32.2)	0.67 (0.29)	
Pain characteristics			
Pain aetiology			
Chronic back pain	31 (52.5)	0.57 (0.25)	0.68
Fibromyalgia	10 (17.0)	0.64 (0.21)	
Other	18 (30.5)	0.56 (0.25)	
Postoperative pain			
Yes	21 (35.6)	0.55 (0.25)	0.53
No	38 (64.4)	0.59 (0.24)	
Peripheral neuropathic pain			
Yes	30 (50.8)	0.53 (0.26)	0.13
No	29 (49.2)	0.62 (0.21)	
Widespread pain			
Yes	29 (49.2)	0.60 (0.20)	0.48
No	30 (50.8)	0.55 (0.28)	
Opioid treatment			
>5 years of opioids exposure			
Yes	20 (33.9)	0.60 (0.24)	0.58
No	39 (66.1)	0.56 (0.24)	
Severe opioid use disorder			
Yes	14 (23.7)	0.60 (0.27)	0.61
No	45 (76.3)	0.57 (0.23)	
Use of tramadol or codeine only			
Yes	4 (6.8)	0.83 (0.33)	*0.02*
No	55 (93.2)	0.56 (0.22)	
Use of oxycodone			
Yes	42 (71.2)	0.56 (0.21)	0.43
No	17 (28.8)	0.61 (0.30)	
Use of transcutaneous fentanyl			
Yes	7 (11.9)	0.52 (0.29)	0.49
No	52 (88.1)	0.58 (0.23)	
Use of oral route only			
Yes	43 (72.9)	0.59 (0.24)	0.44
No	16 (27.1)	0.53 (0.23)	
Use of slow-release only			
Yes	10 (16.9)	0.51 (0.24)	0.39
No	49 (83.1)	0.59 (0.24)	
High daily dose (≥100 morphine milligram equivalent mg/24 hours)			
Yes	44 (74.6)	0.53 (0.19)	*0.02*
No	15 (25.4)	0.70 (0.32)	
Current use of benzodiazepine			
Yes	30 (50.8)	0.57 (0.26)	0.81
No	29 (49.2)	0.58 (0.22)	
Current use of gabapentinoid			
Yes	29 (49.2)	0.53 (0.22)	0.21
No	30 (50.8)	0.61 (0.25)	
Current use of antidepressant			
Yes	46 (78.0)	0.55 (0.25)	0.18
No	13 (22.0)	0.65 (0.19)	
Mean dose (SD) of ketamine in mg/kg/24 hours	0.31 (0.15)	−0.0283	0.83
Mean weight (SD) in kg	74.25 (19.63)	0.1963	0.14

* P value compares the proportion of initial decrease between groups of each categorical covariate or tests the correlation coefficient with the proportion of initial decrease for continuous covariates.

† One-factor analysis of variance for categorical covariates, and by Pearson correlation coefficient or non-parametric Spearman’s rank correlation coefficients for continuous covariates.

corr, correlation coefficient; MME, Morphine Milligram Equivalent; n, number; SD, Standart Deviation.

### Factors associated with failure to sustain opioid tapering at 3 months

Low initial opioid tapering was associated with failure to sustain at 3 months (table 3). On the contrary, widespread pain was associated with less failure to sustain.

**Table 3:** Bivariate analysis for failure to sustain opioid dose decrease at 3 months

	All patients	Sustained tapering	Failure to sustain tapering	P value†
Sample size, n	58	43	15	
Mean age (SD) in years	48.43 (10.52)	47.64 (8.97)	50.70 (14.22)	0.34
Mean weight (SD) in kg	74.31 (19.80)	75.30 (19.90)	71.47 (19.89)	0.56
Female sex, n (%)	38 (65.5)	27 (62.8)	10 (66.7)	0.79
Non-opioid substance use disorder, n (%)	28 (47.5)	24 (55.8)	4 (26.7)	0.05
Tobacco, n (%)	25 (42.4)	21 (48.8)	4 (26.7)	0.23
Alcohol, n (%)	7 (11.9)	6 (14.0)	1 (6.7)	0.66
Cannabis, n (%)	6 (10.2)	5 (11.6)	1 (6.7)	1.00
History of mental disorder, n (%)	40 (67.8)	29 (67.4)	11 (73.3)	0.67
Pain characteristics				
Pain aetiology, n (%)				
Chronic back pain	30 (51.7)	21 (48.8)	9 (75.0)	0.11
Fibromyalgia	10 (17.3)	10 (23.3)	–	
Other	18 (31.0)	12 (27.9)	6 (25.0)	
Postoperative pain, n (%)	21 (35.6)	15 (34.9)	6 (40.0)	0.72
Peripheral neuropathic pain, n (%)	30 (50.9)	19 (44.2)	10 (66.7)	0.13
Widespread pain, n (%)	29 (49.2)	25 (58.1)	3 (20.0)	0.01*
Opioid treatment				
>5 years of opioid exposure, n (%)	20 (33.9)	15 (34.9)	5 (33.3)	0.91
Severe opioid use disorder, n (%)	14 (23.7)	10 (23.3)	3 (20.0)	1.00
Use of tramadol or codeine only, n (%)	4 (6.9)	3 (7.0)	1 (6.7)	1.00
Use of oxycodone, n (%)	42 (71.2)	31 (72.1)	10 (66.7)	0.69
Use of transcutaneous fentanyl, n (%)	7 (11.9)	4 (9.3)	3 (20.0)	0.36
Use of oral route only, n (%)	43 (72.9)	32 (74.4)	10 (66.7)	0.74
Use of slow release only, n (%)	10 (17.0)	6 (14.0)	4 (26.7)	0.27
High daily dose (≥100 morphine milligram equivalent, mg/24 hours)	44 (74.6)	33 (76.7)	10 (66.7)	0.50
Mean initial opioid decrease (SD), in per cent	58.1 (23.6)	64.4 (19.4)	40.1 (25.9)	<0.001*
Concomitant use of benzodiazepine	30 (50.9)	22 (51.2)	8 (53.3)	0.89
Concomitant use of gabapentinoid	29 (49.2)	21 (48.8)	7 (46.7)	0.89
Consomitant use of antidepressant	46 (78.0)	35 (81.4)	10 (66.7)	0.29
Mean dosage of ketamine (SD), in mg/kg/24 hours	0.31 (0.14)	0.30 (0.14)	0.32 (0.15)	0.70

* The p value compares the subjects with a sustained decrease at 3 months with those who failed to sustain tapering.

† The χ2 tests were used for categorical covariates (or Fisher’s exact test if conditions of application were not fulfilled) and t-tests for continuous covariates (or Wilcoxon-Mann-Whitney in case of non-normal distribution or heterogeneity of variances).

MME, Morphine Milligram Equivalent; n, number; OUD, Opioid-Use Disorder; SD, Standart Deviation.

## Discussion

In this pragmatic real-world study, our findings suggest a potential benefit of adding a single infusion of low-dose ketamine during a 5-day hospitalisation to initiate opioid tapering among long-term opioid users with OUD in the context of CP. Our results are of interest because this population is frequent in the context of CP under long-term opioid treatment: treatment failure (ie, opioids were not efficient in relieving pain anymore), for which outpatient progressive opioid tapering failed, and with comorbid OUD. Opioid reductions appear to be maintained over time. Single infusions of low-dose ketamine were well tolerated while minimising withdrawal symptoms.

In the specific context of CP, several interventions have been described as being efficient at reducing opioid dose requirements or allowing opioid cessation: pain self-management programmes, transcutaneous electrical nerve stimulation or even spinal cord stimulation.^24^,^28^ Patients followed in our CP centre received pain self-management counseling or other personalised pain management strategies. In our study, we selected outpatients who failed progressive opioid tapering. All patients were willing to taper opioids during a 5-day hospitalisation with a ketamine infusion. During the hospitalisation, they were closely monitored, and global strategy was improved (including psychological counseling, relaxation, transcutaneous electrical therapy and/or adapted physical activity). In the absence of a control group to compare, it remains unsure if ketamine has potentialised this holistic approach. Compared with the work of Darnall and colleagues (2018), which presented the changes in opioid doses in an outpatient tapering protocol for patients followed in a community pain clinic, our results illustrated a more significant opioid dose decrease and a lower dropout rate with the initial help of ketamine during a short-hospitalisation.^7^ However, in their study, it was unclear if patients were having comorbid OUD.

In the context of CP, the aim of care is to propose a global approach to improve the quality of life and the burden of symptoms. In CP, ‘pain’ no longer plays the role of an alarm, and dysregulation of pain integration occurs at different levels with hypersensitisation.^29^ Long-term use of opioids contributes to these mechanisms. It is recommended to reduce the opioid dose as much as possible.^5^ Unfortunately, the high prevalence of OUD in CP often makes this a challenging exercise.^30^ The main objective of this type of tapering is to limit the hyperalgesia phenomena observed in some patients and to limit the iatrogeny. This paradigm should be distinguished from acute pain and cancer pain, mainly due to excessive nociception, where decreasing pain is necessarily a relevant objective.

In our study, the benefit of ketamine that was observed could be mediated by several pharmacological pathways.^31^ Long-term use of opioids is associated with tolerance, which leads to a gradual escalation of the dose. This increase is related to three main adaptive patterns: (1) the ‘affective effect’ of opioids, where the experience of pain relief activates the reward system,^32^ (2) pharmacological tolerance leading to a decrease in analgesic efficacy^33^ and (3) the gradual onset of opioid-induced hyperalgesia.^33 34^

In our results, psychological comorbidities such as depression and anxiety disorders were identified as potential barriers to ketamine efficacy. Anxiety, depression and other mood disorders are highly prevalent among patients with CP, and even more so among long-term opioid users.^35^ In our study, more than 70% of the population suffered from anxiety or depressive disorders. Depressed patients are more likely to continue long-term opioid use, indicating potential misuse to treat insomnia and anxiety.^36 37^ Interestingly, the antidepressant effect of ketamine involves opioid receptors and may also be explained through anti-inflammatory action.^38 39^ However, the low ketamine doses we used might not have been sufficient to decrease the opioid dose in our population compared with people without any psychiatric disorders. This is consistent with data on the antidepressant effect of ketamine at higher doses (0.5 mg/kg).^40^ We hypothesise that opioid tapering is more difficult in this comorbid subpopulation due to the discomfort generated. This is consistent with a recent study that highlighted that patients who use opioids for a non-analgesic effect (eg, anxiolysis, relaxation and insomnia) were more likely to experience daily withdrawal symptoms.^41^

Our results suggest that alcohol use disorder was associated with a more effective benefit of ketamine on opioid dose reduction. Alcohol use disorders are known to be associated with more intense opioid withdrawal symptoms.^42^ Given fewer opioid withdrawal symptoms with ketamine, that is consistent with that noted in other substance use disorders.^43^ Ketamine is increasingly used in the treatment of alcohol and cocaine disorders, for example.^44 45^ This drug might play a specific role in physiological patterns of addiction. Various mechanisms are currently under investigation: neuroplasticity, neurogenesis, action on the serotoninergic system and memory integration of the reward circuits.^46^ This is consistent with the fact that the reward circuit mechanisms remain functional in the context of long-term opioid use.^47^ It should also be emphasised that in our study, patients with alcohol use disorders might have substituted opioids with alcohol. However, this was not measured, and we were unable to get this information a posteriori.

Regarding opioid tolerance, our results indicate that ketamine might be helpful to quickly taper opioids. Rapidly lowering opioid doses with the help of ketamine could promote the remobilisation of opioid receptors with renewed analgesic potential.^48^ However, tolerance mechanisms are complex and remain to be elucidated.^49^

Lastly, the antihyperalgesic action caused by the NMDA-antagonist effect of ketamine is well documented.^13 50^ In our study, patients with generalised pain showed better sustainment of opioid tapering at 3 months. Widespread pain, regardless of the aetiology, suggests central sensitisation potentially through opioid-induced hyperalgesia. Such hyperalgesia might be predictive of ketamine efficacy in facilitating tapering. It could also be hypothesised that, in the context of hyperalgesia, opioid tapering is facilitated by the fact that it potentially improves the ‘pain’ symptom. In contrast to studies that seek to establish the analgesic potency of ketamine, we propose lower doses of ketamine to help with opioid tapering in the context of CP and OUD. These low doses appear to limit the risk of adverse effects.^17^

Our study has limitations. First, it is a retrospective study and the number of people lost to follow-up was significant. Physical and psychological symptoms were not systematically assessed, neither at the beginning nor during follow-up. This constitutes a significant bias that precludes a causal argument and accounts for the potential concrete benefit of tapering opioids. All patients were volunteers and agreed to participate in this protocol. This might contribute to the success of our strategy. In addition, we had no control group, which would have allowed us to highlight a possible benefit of our approach compared with standard progressive tapering. Our findings could have been influenced by various factors related to the multidisciplinary approach and the effects of being hospitalised. Maintenance over time may be related to other factors such as psychoeducation about CP and OUD or close follow-up after discharge. Prospective and comparative studies are needed to validate our observations and clarify the presumed clinical benefit of ketamine.

## Conclusions

Our study suggests ketamine may be helpful in the initiation of opioid tapering in the context of CP with OUD. Ketamine was administered at a very low dose during a 5-day hospitalisation where patients benefited from a multidisciplinary approach. Opioid tapering appeared to maintain itself over time, but these results are less certain. Ketamine was well tolerated and patients did not present significant withdrawal symptoms during the initiation of tapering.

## Data Availability

No data are available.
